# The HDAC Inhibitor Butyrate Impairs β Cell Function and Activates the Disallowed Gene Hexokinase I

**DOI:** 10.3390/ijms222413330

**Published:** 2021-12-11

**Authors:** Stephanie Bridgeman, Gaewyn Ellison, Philip Newsholme, Cyril Mamotte

**Affiliations:** Curtin Health Innovation Research Institute and Curtin Medical School, School of Molecular and Life Sciences, Curtin University, Bentley, WA 6102, Australia; stephanie.allen@postgrad.curtin.edu.au (S.B.); gaewyn.ellison@curtin.edu.au (G.E.); philip.newsholme@curtin.edu.au (P.N.)

**Keywords:** HDAC inhibitors, butyrate, diabetes, insulin secretion, beta cell

## Abstract

Histone deacetylase (HDAC) inhibitors such as butyrate have been reported to reduce diabetes risk and protect insulin-secreting pancreatic β cells in animal models. However, studies on insulin-secreting cells in vitro have found that butyrate treatment resulted in impaired or inappropriate insulin secretion. Our study explores the effects of butyrate on insulin secretion by BRIN BD-11 rat pancreatic β cells and examined effects on the expression of genes implicated in β cell function. Robust HDAC inhibition with 5 mM butyrate or trichostatin A for 24 h in β cells decreased basal insulin secretion and content, as well as insulin secretion in response to acute stimulation. Treatment with butyrate also increased expression of the disallowed gene hexokinase I, possibly explaining the impairment to insulin secretion, and of *TXNIP*, which may increase oxidative stress and β cell apoptosis. In contrast to robust HDAC inhibition (>70% after 24 h), low-dose and acute high-dose treatment with butyrate enhanced nutrient-stimulated insulin secretion. In conclusion, although protective effects of HDAC inhibition have been observed in vivo, potent HDAC inhibition impairs β cell function in vitro. The chronic low dose and acute high dose butyrate treatments may be more reflective of in vivo effects.

## 1. Introduction

Butyrate is a short-chain fatty acid (SCFA) produced by gut microbial fermentation of resistant starch that is of interest as a therapeutic to protect against type 2 diabetes and the metabolic syndrome [1]. Butyrate alters gene expression by inhibiting histone deacetylases (HDACs), increasing the epigenetic mark of histone acetylation, which is generally associated with active gene expression. Butyrate also binds and activates the G protein-coupled (GPR) free fatty acid receptors (FFAR) FFAR2 and FFAR3, which can influence metabolism, particularly in the intestine, where this action promotes the release of gut hormones such as GLP-1, known to enhance insulin secretion [2]. In animal models of high fat diets, butyrate has been reported to reduce fasting insulin and glucose and to improve glucose and insulin tolerance [3,4,5,6]. Furthermore, it has been reported to reduce β cell apoptosis and prevent pancreatic islet hyperplasia in animal models of diabetes [5,7]. In an ex vivo study, islets from mice treated with butyrate had increased glucose-stimulated insulin secretion (GSIS) with reduced basal insulin secretion [5].

The in vitro story appears to be more complex. Insulin-secreting cell lines treated with butyrate reportedly demonstrated defective GSIS. In particular, a study utilising rat clonal BRIN-BD11 beta cells reported that 72 h butyrate treatment reduced both basal and glucose-stimulated insulin secretion, while also reducing cellular insulin content [8]. However, cell viability was also significantly reduced, indicating this prolonged treatment regime was toxic to the cells. Conversely, two studies of RIN-m5F cells, which are usually poorly responsive to glucose, reported that butyrate increased insulin secretion at both high and low glucose concentrations [9,10]. This was associated with increased activity of glucokinase (high Km hexokinase), as well as low Km hexokinases, which operate at high (physiological) and low glucose concentrations, respectively [9]. This indicates increased glucose sensing at both low and high glucose concentrations, which could result in hyperinsulinemia and affect blood glucose in an in vivo context. These prior studies on BRIN-BD11 and RIN-m5F cells are limited by the use of toxic, prolonged butyrate treatments [8] or the use of less-responsive cell lines [9,10], respectively. Furthermore, Yamato [11] found the potent HDAC inhibitor trichostatin A (TSA) increased basal and decreased stimulated insulin secretion in MIN6 cells, while downregulating important β cell identity genes Pdx1 and MafA, suggesting that the cells were becoming dedifferentiated.

We have previously shown that following 24 h treatment with 5 mM sodium butyrate and 2.5 µM TSA, the HDAC activity of BRIN-BD11 cells is reduced to 25% and to non-detectable levels, respectively, and that butyrate increases global H3K9 and H4K8 acetylation in these cells in a dose-dependent manner [12]. In this study, we investigated the effects of both chronic and acute butyrate exposure on insulin secretion, utilising BRIN-BD11 cells. This included exploration of effects on targeted genes implicated in β cell function and identity and examination of specific histone modifications and the use of TSA to determine whether effects are due to HDAC inhibition.

## 2. Results

### 2.1. Butyrate Is Non-Toxic to BRIN-BD11 Cells

**Figure 1:**
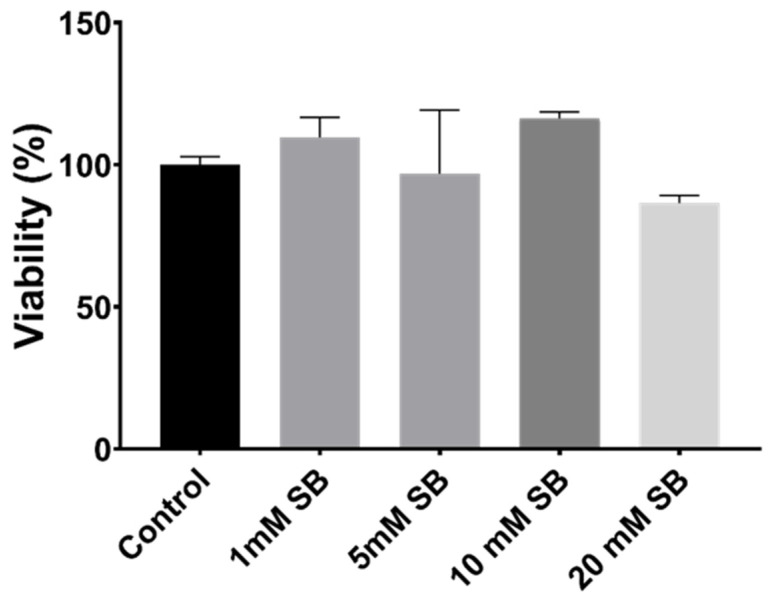
The effects of 24 h butyrate treatment on cell viability, as determined by the alamarBlue^®^ assay.

Treatment of BRIN-BD11 cells with sodium butyrate for 24 h had no significant influence on the viability of BRIN-BD11 cells; even at the very high concentration of 20 mM, cell viability was close to 90% and did not differ significantly from control cells (Figure 1). At 1, 5 and 10 mM butyrate concentrations, viability remained above 95%.

### 2.2. HDAC Inhibition Impairs Insulin Secretion and Reduces Insulin Content in BRIN-BD11 Cells

**Figure 2:**
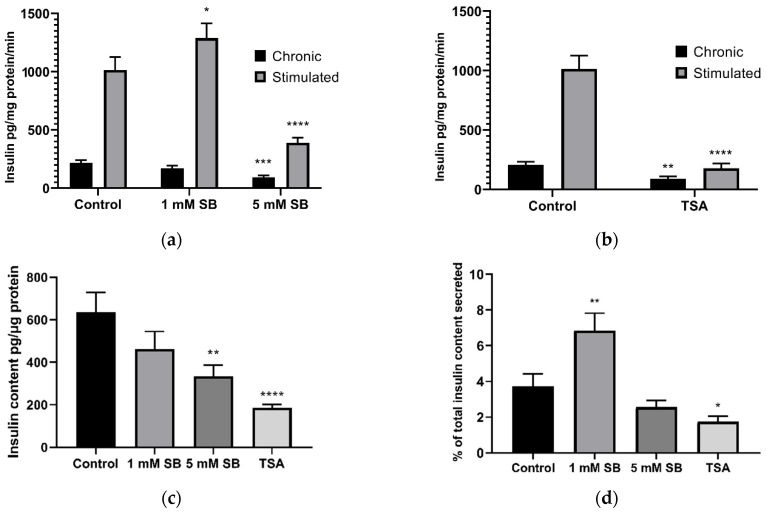
Insulin secretion following 24 h treatment with butyrate and 2.5 µM TSA in BRIN-BD11 cells. The effect of 24 h exposure to (**a**) sodium butyrate (SB) and (**b**) 2.5 µM trichostatin A (TSA) on 24 h (chronic) insulin secretion and in response to acute stimulation by 16.7 mM glucose and 10 mM alanine for 20 min (stimulated); (**c**) the effect of 24 h treatment of sodium butyrate and TSA on BRIN-BD11 cellular insulin content; (**d**) the percentage of total insulin secreted in response to stimulation per the insulin content of unstimulated cells. Results are means combined from at least 3 independent experiments + SEM. * *p* < 0.05, ** *p* < 0.01, *** *p* < 0.001, **** *p* < 0.0001 compared to the relative untreated control.

Treatment of BRIN-BD11 cells with 5 mM sodium butyrate (Figure 2a) and 2.5 µM TSA (Figure 2b) reduced chronic insulin secretion into culture media over the 24 h treatment period, conducted in the present work in media containing 11.1 mM glucose, as is standard [13]. Subsequent acute stimulation with 16.7 mM glucose and 10 mM alanine (a widely accepted positive control condition) [14] caused significant increases in insulin secretion in control cells and in cells treated for 24 h with 1 mM sodium butyrate, but this response was significantly blunted in cells treated with 5 mM sodium butyrate or 2.5 µM TSA for 24 h. The insulin content of these cells was also reduced (Figure 2c). The percentage of cellular insulin content secreted by cells acutely stimulated with glucose and alanine was also decreased by TSA (Figure 2d); thus, the reduction in insulin secretion was not completely accounted for by a decrease in the insulin content of the cells. By contrast, 1 mM sodium butyrate significantly increased the percentage of insulin secreted.

### 2.3. Acute Exposure to Butyrate Synergises Insulin Secretion by Secretagogues

**Figure 3:**
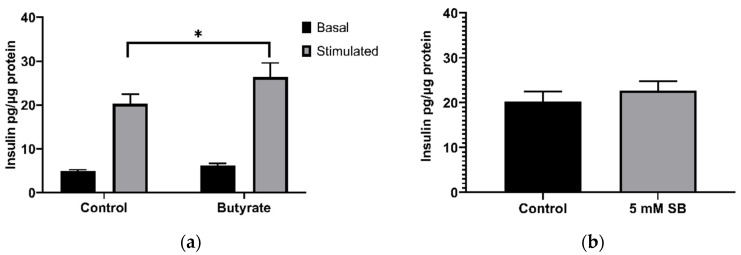
Acute effects of sodium butyrate on insulin secretion in BRIN-BD11 cells. (**a**) Acute effects of 20 min exposure to 5 mM sodium butyrate with (stimulated) or without (basal) 10 mM alanine and 16.7 mM glucose; (**b**) effect of 40 min treatment with 5 mM sodium butyrate followed by 20 min stimulation with 10 mM alanine and 16.7 mM glucose. Results represent the mean combined from three independent experiments, with error bars representing SEMs. * *p* < 0.05.

The previous results demonstrated that 24 h treatment of BRIN-BD11 cells with 5 mM sodium butyrate impaired their ability to respond to acute stimulation by glucose and alanine. However, in the absence of any prior treatment with butyrate, inclusion of 5 mM butyrate in the stimulation media enhanced the secretory response to alanine and glucose (Figure 3a). Butyrate also tended to increase insulin secretion in the absence of alanine and glucose; however, this was not significant (*p* = 0.09). To determine whether this may be due to effects of HDAC inhibition on gene expression, cells were treated with butyrate for 40 min, before addition of the stimulation media without butyrate. However, this short-term butyrate treatment (40 min) failed to significantly enhance stimulated insulin secretion (*p* = 0.43) (Figure 3b). The lack of stimulation following the removal of butyrate suggests that the acute stimulatory effect is unlikely to be due to epigenetic effects and may instead be due to effects mediated via FFAR binding and activation, or due to metabolism of butyrate as an energy source.

### 2.4. Butyrate Upregulates the Disallowed Gene Hexokinase I in BRIN-BD11 and HepG2 Cells

**Figure 4:**
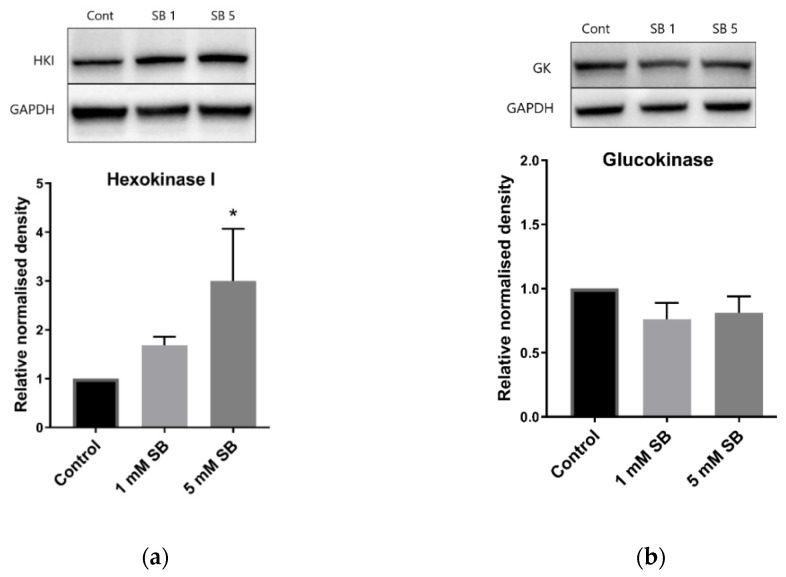
BRIN-BD11 immunoblots of hexokinases. Relative protein levels of (**a**) hexokinase I and (**b**) glucokinase in BRIN-BD11 cells following treatment with 1 mM or 5 mM sodium butyrate for 24 h. The graph represents the mean combined density readings as normalised to GAPDH from three independent experiments, with error bars representing SEMs. * *p* < 0.05.

**Figure 5:**
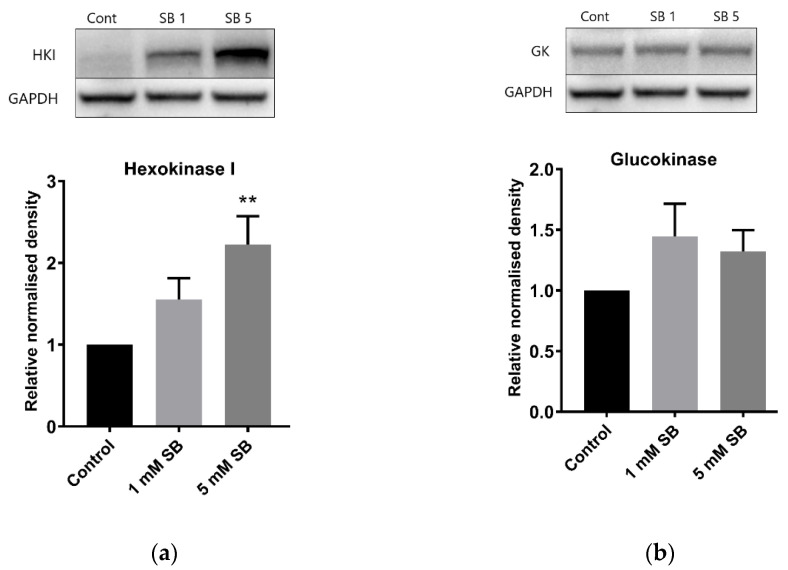
HepG2 immunoblots of hexokinases. Relative protein levels of (**a**) hexokinase I and (**b**) glucokinase in HepG2 cells following treatment with 1 mM or 5 mM sodium butyrate for 24 h. The graph represents the mean combined density readings as normalised to GAPDH from three independent experiments, with error bars representing SEMs. ** *p* < 0.01.

**Figure 6:**
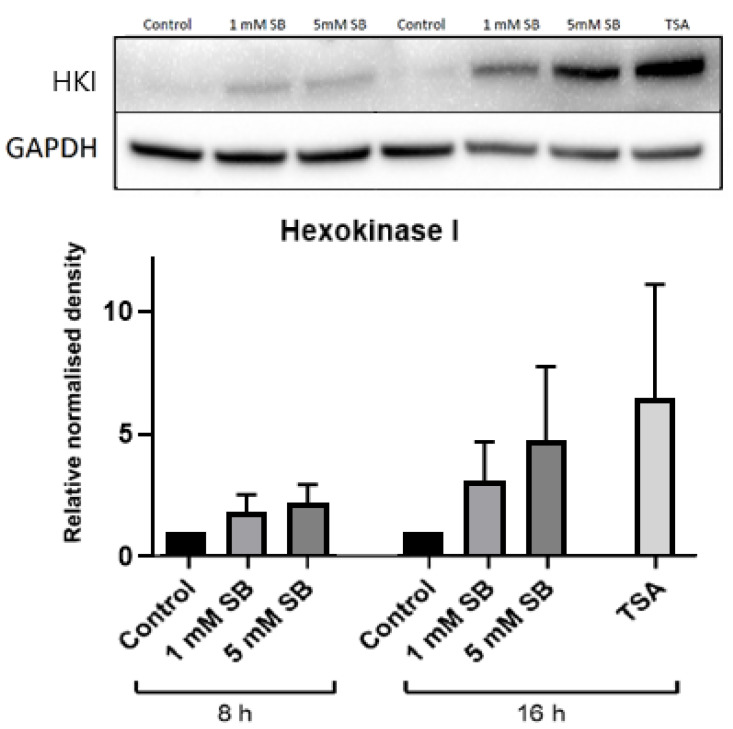
Time-course immunoblot for hexokinase I. Relative protein levels of hexokinase I in HepG2 cells following treatment with 1 mM or 5 mM sodium butyrate for 8 or 16 h or with 2.5 µM TSA for 16 h. The graph represents the mean combined density readings as normalised to GAPDH from three independent experiments, with error bars representing SEMs.

Hexokinases phosphorylate glucose and other hexoses, and for glucose this creates glucose 6-phosphate in the key first step of cellular glucose utilisation. Hexokinase I (*HK1*), which has a low Km, is usually silenced in β cells and liver cells in favour of glucokinase, which has a higher Km [15], although some residual expression is a feature of BRIN-BD11 cells [16]. However, 24 h exposure to 5 mM butyrate caused a substantial increase in *HK1* levels in both BRIN-BD11 cells and liver HepG2 cells (Figure 4a and Figure 5a) but had no effect on glucokinase (Figure 4b and Figure 5b). Signs of increased expression of *HK1* in HepG2 cells were apparent at 8 h of exposure to 5 mM butyrate and with 16 h treatment of TSA, although this was not statistically significant as the degree of upregulation varied between replicate experiments, reflected by the large SEMs (Figure 6).

### 2.5. Butyrate Significantly Alters the Expression of Genes Implicated in β Cell Function and Identity

**Figure 7:**
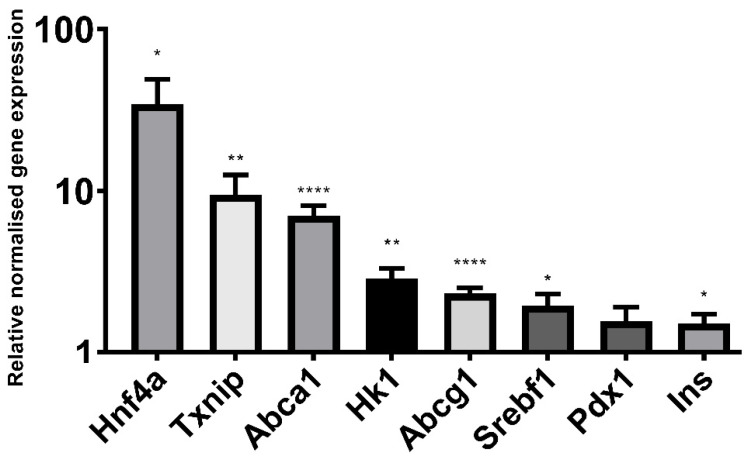
qPCR of glucose metabolism genes. Relative gene expression in BRIN-BD11 cells treated with 5 mM sodium butyrate compared to the untreated control (value of 1). Gene expression is normalised to reference genes *Actb* and *Rpl13a.* Results represent the mean combined from three independent experiments, with error bars representing SEMs. * *p* < 0.05, ** *p* < 0.01, **** *p* < 0.0001.

Sodium butyrate (5 mM) had a significant effect on the mRNA expression of numerous genes that have been linked to β cell function, as determined by RT qPCR (Figure 7). Hexokinase I was upregulated at the mRNA level in addition to that previously described at the protein level (Figure 4 and Figure 5). The important transcription factors for β cell development and identity, *Pdx1* (*p* = 0.06) and *Hnf4a,* were also upregulated, as was the insulin gene itself and the cholesterol transporter genes *Abca1* and *Abcg1*, both of which are important to β cell function and insulin secretion [17]. *Txnip,* which encodes thioredoxin-interacting protein and has been implicated in β cell apoptosis in T2D [18], was upregulated, as was *SREBF1*, which encodes sterol regulatory element-binding protein 1, primarily known for its role in fatty acid synthesis but also known to impair insulin secretion [19].

### 2.6. How Butyrate Activates HK1 Expression Remains Unclear

**Figure 8:**
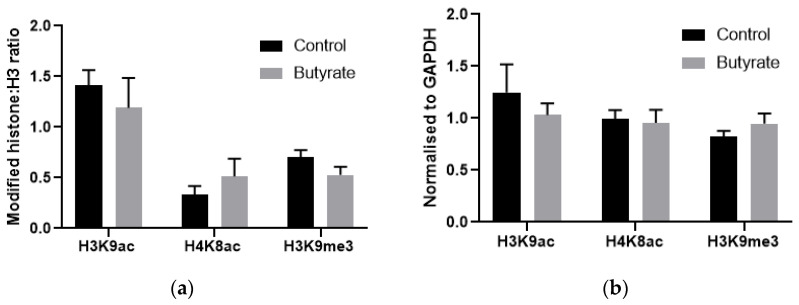
ChIP-qPCR results in proximity to *HK1* normalised to (**a**) total H3 or (**b**) control gene values from HepG2 cells treated with 5 mM butyrate for 24 h. Results represent the mean combined from four independent experiments, with error bars representing SEMs.

Chromatin immunoprecipitation (ChIP) was used to examine histone acetylation in proximity to *HK1* in HepG2 cells. HepG2 cells were used in this experiment rather than BRIN-BD11 cells due to the residual expression of *HK1* in these cells, possibly making any changes more difficult to detect. A region in intron 1 was chosen, as this region has a strong H3K9ac peak in other cell types but not in HepG2 cells, suggesting it may be important to the activation and expression of *HK1*. However, butyrate had no significant effect on H3K9 acetylation or trimethylation, or on H4K8 acetylation in this region, when normalised to either total H3 or to the control gene GAPDH (Figure 8). How butyrate activates *HK1* expression therefore remains unclear.

## 3. Discussion

Potent inhibition of HDAC activity in BRIN-BD11 cells over 24 h with 5 mM sodium butyrate or 2.5 μM TSA significantly impaired both 24 h insulin secretion and secretion in response to acute (20 min) stimulation with well-established potent secretagogues. This is likely due to defects in both insulin production and secretion, since insulin content was decreased by 24 h HDAC inhibition, and the percentage of total insulin content secreted was also lower in cells subject to potent HDAC inhibition. It should be noted that insulin secretion was not increased, and in fact was decreased, during the treatment period, so the reduced content was not due to high levels of insulin secretion depleting stores. As insulin mRNA was increased by 5 mM butyrate, it is unlikely that altered insulin gene expression contributed to the reduced cellular insulin content. It is possible, however, that insulin content may have been affected by influences on translation, processing, or degradation. For example, the insulin ELISA used in this study only recognises mature insulin and thus would not detect changes to the precursors preproinsulin and proinsulin [20].

The activation of hexokinase I expression, seen both in this study and in studies of butyrate-treated RINm5F insulin-secreting cells [9,10], may contribute to the detrimental effects of butyrate on both insulin content and GSIS. Glucokinase has a low affinity for glucose, and thus is only active when glucose concentrations are high, whereas hexokinase I is silenced in healthy β cells as it phosphorylates glucose at low concentrations, which is inappropriate in insulin-secreting cells [15]. Furthermore, unlike glucokinase, low Km hexokinases such as hexokinase I are inhibited by their product, thereby limiting their activity in high-glucose environments. Therefore, hexokinase I expression would result in inappropriate responses to both high and low glucose. Feeding hamsters high sucrose diets has been found to increase islet hexokinase I expression, leading to hyperinsulinaemia [21], and hexokinase I expression was found to increase with age, obesity and diabetes development in rat islets [22]. While hexokinase I expression has been associated with β cell dedifferentiation [23], the increased expression of transcription factors relevant to β cell identity, such as *Pdx1* and *Hnf4a*, as well as the insulin gene itself, suggest that more generalised dedifferentiation has not occurred.

Exposing islets to high glucose (27 mM) for 48 h has been shown to impair insulin secretion [24], associated with an increase in the proinsulin:insulin ratio [25]. Although cellular insulin content is decreased by glucose overstimulation, this only partially explains the impaired insulin secretion [26]. It seems plausible that increased glucose sensing due to hexokinase activity could have similar effects, especially as an increase in the proinsulin:insulin ratio would explain the discord between increased insulin mRNA but decreased insulin content seen in butyrate treated cells. This is comparable to the glucotoxicity thought to occur in T2D, where prolonged hyperglycaemia and subsequent demand for insulin leads to a decline in β cell function and an eventual increase in β cell apoptosis, possibly due to oxidative stress [27].

To explore the mechanism by which HDAC inhibitors increase hexokinase expression, we examined histone acetylation in proximity to the *HK1* gene in HepG2 cells using ChIP. Using ENCODE data [28] an H3K9ac site in proximity to intron 1 of *HK1* with low peak strength in HepG2 cells compared to other cell types was identified. However, ChIP experiments found no change in H3K9 or H4K8 acetylation in this region in butyrate-treated HepG2 cells. Thus, the exact mechanism by which butyrate upregulates hexokinase I remains unknown. Interestingly, it has been shown that the repression of hexokinase I in healthy β cells is epigenetically controlled; Dhawan et al. [29] demonstrated increased DNA methylation of the *Hk1* promoter in β cells from adult mice compared to immature β cells from neonatal mice, with increased binding of the DNA methyltransferase DNMT3A in this region. Histone modifications, namely decreased H3K9 acetylation and increased H3K27 methylation, have been found to play a role in the repression of two other genes disallowed in β cells, *Mct1* and *Ldha* [30]. ChIP-seq across the *Hk1* locus in both β cells and hepatocytes could elucidate the mechanism by which HDAC inhibition activates hexokinase I expression.

We also noted increased expression of thioredoxin-interacting protein (*Txnip*) with butyrate treatment. TXNIP inhibits thioredoxin, a thiol oxidoreductase that reduces oxidised proteins produced by reactive oxygen species [18]. The increase in *Txnip* mRNA thus provides further evidence that HDAC inhibition can increase oxidative stress in β cells; TSA has also been previously found to increase reactive oxygen species in insulin-secreting cells [11]. In β cells, TXNIP is strongly upregulated by glucose and induces apoptosis. TXNIP has been implicated as the link between chronic hyperglycaemia and β cell death [18], and elevated *TXNIP* mRNA has been found in islets isolated from subjects with diabetes [31]. While the observed increase in *Txnip* mRNA could be due to increased glucose sensing as a result of hexokinase I expression, there is also evidence that *TXNIP* is epigenetically controlled. Glucose increases histone acetylation in proximity to the *TXNIP* gene and knockdown or inhibition of the histone acetyltransferase p300 ameliorates the glucose-induced TXNIP upregulation. The increased *Txnip* expression could therefore be a result of HDAC inhibition and subsequent increased histone acetylation; numerous other studies have found that various HDAC inhibitors increase TXNIP expression [11,32,33,34].

Although 24 h treatment with butyrate was detrimental to insulin secretion, acute exposure to butyrate enhanced the secretory response of BRIN-BD11 cells to alanine and glucose. In agreement with this, a historical study by Manns et al. [35] in 1967 reported that infusion of butyrate directly into the pancreatic artery of sheep resulted in a marked increase in plasma insulin within 3 min. More recently, Lin et al. [36] found increased plasma insulin 10 min after orally administering sodium butyrate to mice. This acute secretion is unlikely to be due to changes in gene expression, i.e., due to HDAC inhibition, as short-term (40 min) butyrate treatment prior to stimulation failed to significantly enhance insulin secretion in our study. Instead, butyrate may act as a secretagogue through FFAR activation or through direct metabolism of butyrate as an energy source. Butyrate is the primary energy source for colonocytes [37], while, in vitro, butyric acid increased GSIS in MIN6 cells but not in FFAR2 knockdown cells, suggesting it may be the actions of butyrate as a FFAR agonist that can enhance insulin secretion [38]. Similar results with other SCFAs were reported by Pingitore et al. [39] and Priyadarshini et al. [40], however, other studies have suggested that FFAR activation impairs insulin secretion [41,42]; thus, uncertainty remains regarding the role of FFARs in insulin secretion. It may be that the stimulatory effects of the 1 mM butyrate after 24 h are also a result of FFAR activation or butyrate metabolism, while the detrimental effects of HDAC inhibition are stronger at higher doses. Conversely, it may be that positive effects on gene expression, such as upregulation of *Pdx1, Hnfa* and insulin, have a greater impact on GSIS than the negative effects of upregulation of *Hk1* and *Txnip* at this lower dose.

This study does have its limitations. In particular, the majority of experiments were performed in a single cell line, and this cell line, BRIN-BD11, is defective in its expression of hexokinase I [16], although HepG2 was used as a secondary cell line to explore effects on HK1. Ideally, confirmatory experiments would be carried out in additional insulin-secreting cell lines or, preferably, isolated islets from animal models, though as discussed in the introduction, an effect in upregulating *HK1* has previously also been shown in the RIN-m5F β cell line. Additionally, ideally ChIP experiments would also be expanded to include butyrate and TSA treated BRIN-BD11 and HepG2 cells across multiple genomic loci.

Butyrate and TSA are known to have poor systemic availability and are rapidly metabolised [43,44,45]. Thus, the treatment times and doses used in this and other in vitro studies are unlikely to reflect the in vivo effects of oral treatment. Most notably, rather than impairing insulin secretion and β cell function, in high fat diet (HFD)-fed rodents, butyrate supplementation reduced fasting glucose [5,6,46,47], decreased β cell hyperplasia [5,47], and reduced signs of inflammatory response in islets [47]. In an ex vivo study of pancreatic islets from mice fed a HFD with or without butyrate supplementation, butyrate increased GSIS by increasing glucose-stimulated (16.7 mM) insulin secretion while reducing basal (2.8 mM glucose) insulin secretion [5]. These in vivo and ex vivo effects may not be a direct effect of HDAC inhibition in the β cell, but rather the result of butyrate affecting other tissues. Butyrate stimulates the release of glucagon-like peptide 1 (GLP-1) from intestinal cells both in vitro and in vivo [48,49], as also observed in a human trial [50], and GLP-1 is an established enhancer of β cell function and insulin secretion [51]. It may also be that cells are exposed to low doses of butyrate for a short time period, and thus the low dose and acute butyrate treatments may be more reflective of in vivo effects than the supraphysiological 24 h treatments.

## 4. Materials and Methods

### 4.1. Creation of Stock Solutions

Sodium butyrate (Sapphire Bioscience, NSW Australia) was dissolved in ultra-pure distilled water to a concentration of 50 mM and stored at −20 °C. Trichostatin A (TSA) (Cayman Chemical, MI, USA) was dissolved in dimethyl sulfoxide (DMSO) (Sigma-Aldrich) to a concentration of 10 mM and stored at −20 °C or at −80 °C for long-term use.

### 4.2. Cell Culture and Treatments

BRIN-BD11 rat insulin-secreting cells were maintained in Roswell Park Memorial Institute (RPMI) media (Sigma-Aldrich, CA, USA), supplemented with 10% foetal bovine serum (FBS) (Fisher Biotec, WA, Australia) and 1% penicillin and streptomycin (Sigma-Aldrich). HepG2 human hepatocellular carcinoma cells were maintained in Dulbecco’s Modified Eagle’s medium: Ham’s F-12 Nutrient Mixture (DMEM:F12 media) (Sigma-Aldrich) supplemented with 10% FBS and 1% penicillin and streptomycin. Cells were maintained in 25 cm^2^ or 75 cm^2^ tissue culture flasks at 37 °C in a humidified incubator equilibrated with 5% CO_2_. All cells tested negative for mycoplasma contamination. Cells were seeded in 6-well plates, 96-well plates, 25 cm^2^ vented tissue culture flasks or 75 cm^2^ vented tissue culture flasks and allowed to recover overnight prior to treatment. All cells were treated in media supplemented with 10% LPDS, as described in Bridgeman et al. [12].

### 4.3. Viability Assays

Viability was determined using alamarBlue^®^ assay (ThermoFisher Scientific, MA, USA). Following 22 h treatment in 96-well plates, alamarBlue^®^ was added to the treatment media and cells were incubated for a further 2 h. Fluorescence was then read using an EnSpire Multimode Plate Reader (PerkinElmer, OH, USA) with an excitation wavelength of 540 nm and an emission wavelength of 590 nm.

### 4.4. Protein Extraction

Cells were scraped from confluent flasks then lysed on ice for 20 min in radioimmunoprecipitation assay (RIPA) lysis buffer (Sigma-Aldrich) containing protease and phosphatase inhibitors (Sigma-Aldrich). The lysate was sonicated 10 times for 10 s, then centrifuged at 14,000 rpm for 10 min and the protein-rich supernatant was removed for protein quantification by the bicinchoninic acid (BCA) method using the Pierce BCA Protein Assay Kit (ThermoFisher Scientific).

### 4.5. Immunoblotting

Equal amounts of protein (20–30 µg depending on sample concentration and amount of protein of interest in the sample) were denatured at 98 °C for 10 min in a solution containing Bolt Sample Reducing Agent (ThermoFisher Scientific) and SDS Sample Loading Buffer (Sigma Aldrich), then fractionated on Bolt™ 4–12% Bis-Tris Plus Gels (ThermoFisher Scientific). Proteins were transferred to nitrocellulose membranes using the iBlot Gel Transfer Device (Invitrogen) and blocked with 3% bovine serum albumin (BSA) (Bovogen, VIC, Australia) in Tris-buffered saline with 0.1% Tween 20 (TBST) for at least 1 h. Membranes were incubated overnight, with primary antibodies (Table 1) diluted in blocking buffer. Membranes were incubated with the secondary antibodies conjugated to horseradish peroxidase (HRP) (Table 1) in blocking buffer for at least 1 h, then washed 3 times with TBST. Immunodetection and quantification were performed using the Amersham ECL Prime Western Blotting Detection Reagent (GE Healthcare Life Sciences, IL, USA) and the Chemi-Doc™ Gel Imaging System (Bio-Rad, CA, USA). Band density was measured with Image Lab software (Bio-Rad) and normalised to the density of the housekeeping protein GAPDH.

### 4.6. Insulin Secretion

#### 4.6.1. Insulin Secretion Assay

Following 24 h treatment with HDAC inhibitors in 96-well plates, media were transferred to a fresh plate, centrifuged to remove any cells, and the supernatant was stored at −80 °C as ‘chronic’ insulin secretion samples. Cells were washed in PBS and incubated for 40 min at 37 °C in Krebs Ringer Bicarbonate Buffer (KRBB, 115 mM NaCl, 4.7 mM KCl, 2.5 mM CaCl_2_, 1.2 mM KH_2_PO_4_, 1.2 mM MgSO_4_·7H_2_O, 24 mM NaHCO_3_, 0.1% HEPES (*v*/*v*), 0.1% BSA (*w*/*v*), pH 7.4) to allow the cells to metabolise any residual glucose. A subset of previously untreated cells was treated for 40 min with 5 mM sodium butyrate in the KRBB during this fasting stage. Subsequently, cells were washed in PBS and incubated for 20 min at 37 °C with 10 mM alanine and 16.7 mM glucose in KRBB to robustly stimulate insulin secretion [52]. A subset of previously untreated cells was given 5 mM sodium butyrate in the stimulation media and a further subset used only KRBB ± 5 mM sodium butyrate to determine if butyrate can act as a secretagogue. Media were then transferred to a fresh plate, centrifuged to remove any cells, and the supernatant was stored at −80 °C for later insulin measurement. RIPA lysis buffer (Sigma-Aldrich) was added to cells for protein quantification using the Pierce BCA Protein Assay Kit (ThermoFisher Scientific). Insulin was assayed by sandwich enzyme-linked immunosorbent assay (ELISA) using an ultrasensitive Insulin ELISA kit (Mercodia, Uppsala, Sweden) as per the manufacturer’s instructions.

#### 4.6.2. Cellular Insulin Content

Following HDAC treatment as described above, unstimulated cells were washed with PBS and incubated with acid ethanol (1.5% HCl in 70% ethanol) overnight at 4 °C. This solution was transferred to a fresh plate and stored at −80 °C for later insulin quantification. RIPA lysis buffer was added to cells for protein quantification as described above.

### 4.7. Gene Expression

#### 4.7.1. RNA Extraction

RNA extraction was performed using the TRI reagent protocol as described by Rio et al. [53] using 1-Bromo-3-chloropropane (BCP) in place of chloroform for phase separation [54]. Treated cells in 6-well plates were washed twice in PBS and 300 µL TRI reagent (Sigma-Aldrich) was added directly to cells. Following mixing, the solution was transferred to 1.5 mL tubes, BCP was added, and tubes were vortexed and incubated at RT for 10 min. Tubes were centrifuged at 14,000 rpm for 15 min at 4 °C and the top aqueous phase carefully transferred to fresh low adherence 1.5 mL tubes. An equal volume of isopropanol was then added and tubes were mixed by inversion and incubated at room temperature for 10 min. The tubes were then centrifuged at 14,000 rpm for 15 min at 4 °C, the supernatant was removed, and the RNA pellet was washed twice with 75% ethanol and allowed to air dry. Pellets were dissolved in nuclease-free water and allowed to equilibrate for 1 h at room temperature. RNA was quantified using the NanoDrop 1000 spectrophotometer (ThermoFisher Scientific), with purity considered sufficient if *A*_260_/*A*_280_ measured between 1.8–2.0. Samples were stored at −80 °C.

#### 4.7.2. Reverse Transcription

Reverse transcription was performed using the SensiFAST™ cDNA Synthesis Kit (Bioline) according to the manufacturer’s instructions. A total of 1 µg of RNA was used per reaction in a 20 µL reaction mix containing reverse transcriptase and a blend of anchored oligo dT and random hexamer primers. Reverse transcription was performed in a Veriti 96-Well Thermal Cycler (Applied Biosystems, MA, USA) with the following program: 25 °C for 10 min (primer annealing), 42 °C for 15 min (reverse transcription), 48 °C for 15 min (reverse transcription of complex RNA), 85 °C for 5 min (reverse transcriptase inactivation), 4 °C hold (cooling). cDNA was diluted in 20 µL ultrapure water and stored at 4 °C.

#### 4.7.3. qPCR

Quantitative real-time PCR (qPCR) was performed using the DNA intercalating fluorescent dye, SYBR-Green [55]. Predesigned KiCqStart^®®^ SYBR^®®^ Green Primers (Sigma-Aldrich, sequences in Table 2) were dissolved in ultrapure water to a concentration of 100 µM and stored at −80 °C. Working primer mixes of 5 µM forward and reverse primers in ultrapure water were stored at 4 °C. SensiFAST SYBR Lo-ROX Mix (Bioline) was used with 200 nM primer mix, except for *Actb and Rpl13a*, where 100 nM primers were used due to the presence of multiple peaks seen on melt curve analysis at 200 nM, a 2 µL cDNA sample template and ultrapure water in a 10 µL reaction mix. qPCR was performed in a CFX Connect Real-Time System (Bio-Rad) with the following program: 95 °C for 2 min, followed by 40 cycles of 95 °C for 15 s, 57–62 °C for 15 s (Table 2) and 72 °C for 1 min. Gene expression was normalised to *Actb* and *Rpl13a* reference genes. Normalised gene expression (∆∆C_q_) was calculated using Bio-rad CFX Manager 3.1 software.

### 4.8. Chromatin Immunoprecipitation

Histone modifications at specific genomic regions was determined by chromatin immunoprecipitation (ChIP) using the abcam ChIP Kit. Cells were trypsinised and equal numbers were lysed in a series of buffers from the ChIP Kit according to the manufacturer’s instructions. The lysate was then sonicated in a buffer containing protease inhibitors 3 times for 10 s so as to shear chromatin. This was then incubated overnight at 4 °C on a rotating rack with ChIP-grade antibodies against H3 (positive control), H3K9ac, H4K8ac and H3K9me3 (Table 3), or frozen as input chromatin. Chromatin–antibody complexes were then precipitated with protein A sepharose beads. Immunoprecipitated chromatin and input chromatin were then purified with the provided DNA purifying slurry followed by the addition of Proteinase K. The slurry was pelleted, and the supernatant was either immediately used for qPCR or frozen at −80 °C.

#### ChIP qPCR

ChIP primers were designed using the protocol from the Bridges Lab (http://bridgeslab.sph.umich.edu, accessed on 30 July 2019) [56]. Genomic regions of interest were determined using data from the Encyclopedia of DNA Elements (ENCODE) project [28]. H3K9ac ChIP-seq data from HepG2 and other cell types from ENCODE were visualised using the UCSC Genome Browser on the human Feb. 2009 (GRCh37/hg19) assembly (http://genome.ucsc.edu, accessed on 30 July 2019) [57]. An H3K9ac peak in proximity to *HK1* with varying peak strength in HepG2 cells compared to other cell types was chosen. For *GAPDH*, used as a positive control, a region with strong H3K9ac peaks in multiple cell types was chosen. DNA of the regions of interest was entered into Primer-BLAST (www.ncbi.nlm.nih.gov/tools/primer-blast, accessed on 30 July 2019) and primers with a PCR product size of 70–150 bp were obtained. Primers were ordered through Sigma-Aldrich fully deprotected and desalted in de-ionized water at a concentration of 100 µM. ChIP primers are listed in Table 4.

qPCR was conducted as for gene expression using an annealing temperature of 63 °C. Relative quantity (∆C_q_) was calculated using Bio-rad CFX Manager 3.1 software.

### 4.9. Statistical Analysis

At least three independent replicates were conducted of each experiment. Statistical significance was determined by analysis of variance (ANOVA) or Student’s *t* test depending on the number of treatments being compared, with results considered significant if *p* < 0.05. The analyses were conducted using GraphPad Prism software.

## Figures and Tables

**Table 1:** Antibodies used in immunoblotting.

Target	Type	Product	Company
GAPDH	Mouse monoclonal	ab8245	abcam
Glucokinase	Rabbit polyclonal	ab37796	abcam
Hexokinase 1	Rabbit monoclonal	2024	Cell Signalling
Goat anti-mouse	IgG H&L (HRP)	ab6789	abcam

**Table 2:** Primers for qPCR experiments in BRIN-BD11 cells.

Gene	Forward Primer	Reverse Primer	Annealing Temperature
*Actb*	AAGACCTCTATGCCAACAC	TGATCTTCATGGTGCTAGG	57–61° ^1^
*Rpl13a*	GCACAAGACCAAAAGAGG	CGCTTTTTCTTGTCATAGGG	57–61° ^1^
*Hk1*	AAACTCTGGGAAACAAAGG	AAACTTGGTCTCAAAGATGC	57°
*Hnf4a*	TGTGTGAGTCTATGAAGGAG	ATGTAGTCATTGCCTAGGAG	61°
*Pdx1*	AAAGGTTACAAACTTGAGCG	AAACAGCTCCCTTTATTCTC	59°
*Srebf1*	AAACCTGAAGTGGTAGAAAC	TTATCCTCAAAGGCTGGG	59°
*Ins1*	AACGTGGTTTCTTCTACAC	TCTCCAGTTGGTAGAGGG	61°
*Abcg1*	GTTATGTTCTTTGATGAGCCC	CCTTGACTTAGGACATAAAGC	57°
*Abca1*	CATCTGAAAAACAGGTTTGG	GGGAGAGAATGCTGAATATC	57°
*Txnip*	CGTCAATACTCCTGACTTAATG	AAATGTCATCACCTTCACAG	61°

^1^ Annealing temperature for reference genes varied to match that of the gene of interest in each assay.

**Table 3:** Antibodies used for ChIP.

Target	Type	Product	Company
H3	Rabbit polyclonal	ab1791	abcam
H3K9ac	Rabbit polyclonal	ab10812	abcam
H4K8ac	Rabbit polyclonal	ab15823	abcam
H3K9me3	Rabbit polyclonal	ab8898	abcam

**Table 4:** Primers used for ChIP qPCR.

Genomic Region	Forward Primer	Reverse Primer
*GAPDH* exon 2	CCACATCGCTCAGACACCAT	CATACGACTGCAAAGACCCG
*HK1* intron 1	AGGTCAAAAAGGTGAGCCCC	CTGCAGTCCAACTCGATGCT

## References

[1] Bridgeman S.C., Northrop W., Melton P.E., Ellison G.C., Newsholme P., Mamotte C.D.S. (2020). Butyrate, generated by gut microbiota, and its therapeutic role in metabolic syndrome. Pharmacol. Res..

[2] McNabney S.M., Henagan T.M. (2017). Short chain fatty acids in the colon and peripheral tissues: A focus on butyrate, colon cancer, obesity and insulin resistance. Nutrients.

[3] Gao Z., Yin J., Zhang J., Ward R.E., Martin R.J., Lefevre M., Cefalu W.T., Ye J. (2009). Butyrate improves insulin sensitivity and increases energy expenditure in mice. Diabetes.

[4] Khan S., Jena G. (2016). Sodium butyrate reduces insulin-resistance, fat accumulation and dyslipidemia in type-2 diabetic rat: A comparative study with metformin. Chem. Biol. Interact..

[5] Matheus V., Monteiro L., Oliveira R., Maschio D.A., Collares-Buzato C.B. (2017). Butyrate reduces high-fat diet-induced metabolic alterations, hepatic steatosis and pancreatic beta cell and intestinal barrier dysfunctions in prediabetic mice. Exp. Biol. Med..

[6] Mollica M.P., Mattace Raso G., Cavaliere G., Trinchese G., De Filippo C., Aceto S., Prisco M., Pirozzi C., Di Guida F., Lama A. (2017). Butyrate regulates liver mitochondrial function, efficiency, and dynamics in insulin-resistant obese mice. Diabetes.

[7] Hu Y., Liu J., Yuan Y., Chen J., Cheng S., Wang H., Xu Y. (2018). Sodium butyrate mitigates type 2 diabetes by inhibiting PERK-CHOP pathway of endoplasmic reticulum stress. Environ. Toxicol. Pharmacol..

[8] Liu H.K., Green B.D., Flatt P.R., McClenaghan N.H., McCluskey J.T. (2004). Effects of long-term exposure to nicotinamide and sodium butyrate on growth, viability, and the function of clonal insulin secreting cells. Endocr. Res..

[9] Fernandez-Mejia C., Davidson M.B. (1993). Effect of sodium butyrate on glucose transport and glucose-phosphorylating enzymes in RIN-m5F cells. Pancreas.

[10] Tiedge M., Lenzen S. (1996). Effects of sodium butyrate on glucose transporter and glucose-phosphorylating enzyme gene expression in RINm5F insulinoma cells. J. Mol. Endocrinol..

[11] Yamato E. (2018). High dose of histone deacetylase inhibitors affects insulin secretory mechanism of pancreatic beta cell line. Endocr. Regul..

[12] Bridgeman S., Northrop W., Ellison G., Sabapathy T., Melton P.E., Newsholme P., Mamotte C.D.S. (2019). Statins do not directly inhabit the activity of major epigenetic modifying enzymes. Cancers.

[13] Carlessi R., Chen Y., Rowlands J., Cruzat V.F., Keane K.N., Egan L., Mamotte C., Stokes R., Gunton J.E., de Bittencourt P.I.H. (2017). GLP-1 receptor signalling promotes β-cell glucose metabolism via mTOR-dependent HIF-1α activation. Sci. Rep..

[14] Liu Z., Jeppesen P.B., Gregersen S., Chen X., Hermansen K. (2008). Dose- and glucose dependent effects of amino acids in insulin secretion from isolated mouse islets and clonal INS-1E Beta-Cells. Rev. Diabet. Stud..

[15] Lemaire K., Thorrez L., Schuit F. (2016). Disallowed and allowed gene expression: Two faces of mature islet beta cells. Annu. Rev. Nutr..

[16] Green A.D., Vasu S., Flatt P.R. (2018). Cellular models for beta-cell function and diabetes gene therapy. Acta Physiol..

[17] Kruit J.K., Wijesekara N., Westwell-Roper C., Vanmierlo T., de Haan W., Bhattacharjee A., Tang R., Wellington C.L., LütJohann D., Johnson J.D. (2012). Loss of both ABCA1 and ABCG1 results in increased disturbances in islet sterol homeostasis, inflammation, and impaired β-Cell function. Diabetes.

[18] Alhawiti N.M., Al Mahri S., Aziz M.A., Malik S.S., Mohammad S. (2017). TXNIP in metabolic regulation: Physiological role and therapeutic outlook. Curr. Drug Targets.

[19] Shimano H., Amemiya-Kudo M., Takahashi A., Kato T., Ishikawa M., Yamada N. (2007). Sterol regulatory element–binding protein-1c and pancreatic β-cell dysfunction. Diabetes Obes. Metab..

[20] Fu Z., Gilbert E.R., Liu D. (2013). Regulation of insulin synthesis and secretion and pancreatic beta-cell dysfunction in diabetes. Curr. Diabetes Rev..

[21] Maiztegui B., Borelli M.I., Massa M.L., Del Zotto H., Gagliardino J.J. (2006). Enhanced expression of hexokinase I in pancreatic islets induced by sucrose administration. J. Endocrinol..

[22] Cockburn B.N., Ostrega D.M., Sturis J., Kubstrup C., Polonsky C.S., Bell G.I. (1997). Changes in pancreatic islet glucokinase and hexokinase activities with increasing age, obesity, and the onset of diabetes. Diabetes.

[23] Weir G.C., Aguayo-Mazzucato C., Bonner-Weir S. (2013). β-cell dedifferentiation in diabetes is important, but what is it?. Islets.

[24] Björklund A., Lansner A., Grill V.E. (2000). Glucose-induced [Ca2+]i abnormalities in human pancreatic islets: Important role of overstimulation. Diabetes.

[25] Bjorklund A., Grill V. (1999). Enhancing effects of long-term elevated glucose and palmitate on stored and secreted proinsulin-to-insulin ratios in human pancreatic islets. Diabetes.

[26] Grill V., Björklund A. (2001). Overstimulation and beta-cell function. Diabetes.

[27] Robertson R.P., Harmon J., Tran P.O., Tanaka Y., Takahashi H. (2003). Glucose toxicity in β-Cells: Type 2 diabetes, good radicals gone bad, and the glutathione connection. Diabetes.

[28] ENCODE Project Consortium (2012). An integrated encyclopedia of DNA elements in the human genome. Nature.

[29] Dhawan S., Tschen S.-I., Zeng C., Guo T., Hebrok M., Matveyenko A., Bhushan A. (2015). DNA methylation directs functional maturation of pancreatic β cells. J. Clin. Investig..

[30] Thorrez L., Laudadio I., Van Deun K., Quintens R., Hendrickx N., Granvik M., Lemaire K., Schraenen A., Van Lommel L., Lehnert S. (2011). Tissue-specific disallowance of housekeeping genes: The other face of cell differentiation. Genome Res..

[31] Bompada P., Atac D., Luan C., Andersson R., Omella J.D., Laakso E.O., Wright J., Groop L., De Marinis Y. (2016). Histone acetylation of glucose-induced thioredoxin-interacting protein gene expression in pancreatic islets. Int. J. Biochem. Cell Biol..

[32] Feingold P.L., Surman D.R., Brown K., Xu Y., McDuffie L.A., Shukla V., Reardon E.S., Crooks D.R., Trepel J.B., Lee S. (2018). Induction of thioredoxin-interacting protein by a histone deacetylase inhibitor, entinostat, is associated with DNA damage and apoptosis in esophageal adenocarcinoma. Mol. Cancer Ther..

[33] Perrone L., Devi T.S., Hosoya K.I., Terasaki T., Singh L.P. (2010). Inhibition of TXNIP expression in vivo blocks early pathologies of diabetic retinopathy. Cell Death Dis..

[34] Elgort M.G., O’Shea J.M., Jiang Y., Ayer D.E. (2010). Transcriptional and translational downregulation of thioredoxin interacting protein is required for metabolic reprogramming during G(1). Genes Cancer.

[35] Manns J., Boda J., Willes R. (1967). Probable role of propionate and butyrate in control of insulin secretion in sheep. Am. J. Physiol..

[36] Lin H.V., Frassetto A., Kowalik E.J., Nawrocki A.R., Lu M.M., Kosinski J.R., Hubert J.A., Szeto D., Yao X., Forrest G. (2012). Butyrate and propionate protect against diet-induced obesity and regulate gut hormones via free fatty acid receptor 3-independent mechanisms. PLoS ONE.

[37] Donohoe D.R., Garge N., Zhang X., Sun W., O’Connell T.M., Bunger M.K., Bultman S.J. (2011). The microbiome and butyrate regulate energy metabolism and autophagy in the mammalian colon. Cell Metab..

[38] Traisaeng S., Batsukh A., Chuang T.-H., Herr D.R., Huang Y.-F., Chimeddorj B., Huang C.-M. (2020). Leuconostoc mesenteroides fermentation produces butyric acid and mediates Ffar2 to regulate blood glucose and insulin in type 1 diabetic mice. Sci. Rep..

[39] Pingitore A., Gonzalez-Abuin N., Ruz-Maldonado I., Huang G.C., Frost G., Persaud S.J. (2019). Short chain fatty acids stimulate insulin secretion and reduce apoptosis in mouse and human islets in vitro: Role of free fatty acid receptor 2. Diabetes Obes. Metab..

[40] Priyadarshini M., Villa S.R., Fuller M., Wicksteed B., Mackay C.R., Alquier T., Poitout V., Mancebo H., Mirmira R.G., Gilchrist A. (2015). An acetate-specific GPCR, FFAR2, regulates insulin secretion. Mol. Endocrinol..

[41] Priyadarshini M., Layden B.T. (2015). FFAR3 modulates insulin secretion and global gene expression in mouse islets. Islets.

[42] Tang C., Ahmed K., Gille A., Lu S., Gröne H.-J., Tunaru S., Offermanns S. (2015). Loss of FFA2 and FFA3 increases insulin secretion and improves glucose tolerance in type 2 diabetes. Nat. Med..

[43] Damaskos C., Garmpis N., Valsami S., Kontos M., Spartalis E., Kalampokas T., Kalampokas E., Athanasiou A., Moris D., Daskalopoulou A. (2017). Histone deacetylase inhibitors: An attractive therapeutic strategy against breast cancer. Anticancer Res..

[44] van der Beek C.M., Bloemen J.G., van den Broek M.A., Lenaerts K., Venema K., Buurman W.A., Dejong C.H. (2015). Hepatic uptake of rectally administered butyrate prevents an increase in systemic butyrate concentrations in humans. J. Nutr..

[45] Miller A.A., Kurschel E., Osieka R., Schmidt C.G. (1987). Clinical pharmacology of sodium butyrate in patients with acute leukemia. Eur. J. Cancer Clin. Oncol..

[46] Mattace Raso G., Simeoli R., Russo R., Iacono A., Santoro A., Paciello O., Ferrante M.C., Canani R.B., Calignano A., Meli R. (2013). Effects of sodium butyrate and its synthetic amide derivative on liver inflammation and glucose tolerance in an animal model of steatosis induced by high fat diet. PLoS ONE.

[47] Li H.-P., Chen X., Li M.-Q. (2013). Butyrate alleviates metabolic impairments and protects pancreatic β cell function in pregnant mice with obesity. Int. J. Clin. Exp. Pathol..

[48] Christiansen C.B., Gabe M.B.N., Svendsen B., Dragsted L.O., Rosenkilde M.M., Holst J.J. (2018). The impact of short-chain fatty acids on GLP-1 and PYY secretion from the isolated perfused rat colon. Am. J. Physiol. Gastrointest. Liver Physiol..

[49] Yadav H., Lee J.H., Lloyd J., Walter P., Rane S.G. (2013). Beneficial metabolic effects of a probiotic via butyrate-induced GLP-1 hormone secretion. J. Biol. Chem..

[50] Roshanravan N., Mahdavi R., Alizadeh E., Jafarabadi M.A., Hedayati M., Ghavami A., Alipour S., Alamdari N.M., Barati M., Ostadrahimi A. (2017). Effect of butyrate and inulin supplementation on glycemic status, lipid profile and glucagon-like peptide 1 level in patients with type 2 diabetes: A randomized double-blind, placebo-controlled trial. Horm. Metab. Res..

[51] Rowlands J., Heng J., Newsholme P., Carlessi R. (2018). Pleiotropic effects of GLP-1 and analogs on cell signaling, metabolism, and function. Front. Endocrinol..

[52] Brennan L., Shine A., Hewage C., Malthouse J.P.G., Brindle K.M., McClenaghan N., Flatt P.R., Newsholme P. (2002). A nuclear magnetic resonance-based demonstration of substantial oxidative L-alanine metabolism and L-alanine-enhanced glucose metabolism in a clonal pancreatic beta-cell line: Metabolism of L-alanine is important to the regulation of insulin secretion. Diabetes.

[53] Rio D.C., Ares M., Hannon G.J., Nilsen T.W. (2010). Purification of RNA using TRIzol (TRI reagent). Cold Spring Harb. Protoc..

[54] Chomczynski P., Mackey K. (1995). Substitution of chloroform by bromo-chloropropane in the single-step method of RNA isolation. Anal. Biochem..

[55] Ponchel F., Toomes C., Bransfield K., Leong F.T., Douglas S.H., Field S.L., Bell S.M., Combaret V., Puisieux A., Mighell A.J. (2003). Real-time PCR based on SYBR-Green I fluorescence: An alternative to the TaqMan assay for a relative quantification of gene rearrangements, gene amplifications and micro gene deletions. BMC Biotechnol..

[56] (2016). RT-PCR Primer Design for ChIP Michigan: The University of Michigan. http://bridgeslab.sph.umich.edu/protocols/index.php/RT-PCR_primer_design_for_ChIP.

[57] Kent W.J., Sugnet C.W., Furey T.S., Roskin K.M., Pringle T.H., Zahler A.M., Haussler D. (2002). The Human Genome Browser at UCSC. Genome Res..

